# Higher Lymph Node Metastasis Rate and Poorer Prognosis of Intestinal-Type Gastric Cancer Compared to Diffuse-Type Gastric Cancer in Early-Onset Early-Stage Gastric Cancer: A Retrospective Study

**DOI:** 10.3389/fmed.2021.758977

**Published:** 2021-12-23

**Authors:** Chao-Tao Tang, Si-Hai Chen

**Affiliations:** Department of Gastroenterology, The First Affiliated Hospital of Nanchang University, Nanchang, China

**Keywords:** lymph node metastasis, Lauren type, survival, early-onset, early gastric cancer

## Abstract

**Background:** The incidence of early-onset gastric cancer (GC) that was diagnosed at <50 years is increasing, but there is a knowledge gap on early-onset early-stage GC (EEGC) that was defined as early-onset GC limited to the mucosa or submucosa. Therefore, we comprehensively analysed the clinical features based on Lauren type.

**Methods:** Logistic and Cox analyses were used to investigate risk factors for lymph node metastasis (LNM) and prognosis, respectively. Propensity score matching (PSM) was used to adjust confounding factors. Protein mass spectrometry analysis was used to explore the molecular mechanism of LNM.

**Result:** Our study included 581 patients with EEGC from the Surveillance, Epidemiology, and End Results (SEER) database and 226 patients with EEGC from our own centre. We identified intestinal type, T1b stage, and tumour size (>3 cm) as risk factors for LNM using SEER and our own data. We also found that the prognosis of patients with intestinal-type EEGC was poorer than patients with diffuse-type EEGC, and T1b stage and positive LNM were hazard factors for survival. After analysing the expression of proteins between positive and negative LNM in the intestinal or diffuse type, we found no similar proteins between these groups. The differentially expressed genes (DEGs) in the intestinal type functioned as epithelial cell signalling in *Helicobacter pylori*. The DEGs in the diffuse type functioned in the tricarboxylic acid cycle (TCA cycle) and oxidative phosphorylation.

**Conclusion:** For EEGC, our study was the first report to demonstrate that the intestinal type was a risk factor for LNM and survival compared to the diffuse type, and the oncogenic expression promoting the occurrence of LNM was different. These findings suggest that clinicians should pay more attention to intestinal-type EEGC than diffuse-type EEGC.

## Introduction

Global cancer statistics reported that gastric cancer (GC) was the fifth most common tumour in incidence in 2020, and is ranked fourth in cancer-related mortality (1). The incidence and mortality of GC steadily declined over the last decade, especially for non-cardia GC. However, a recent notable finding was that the incidence of GC increased in young patients aged ≤50 years (2, 3). Several studies named cases diagnosed at >50 years as early-onset GC (4–6). Lauren classification was proposed in 1965, and it has been widely used in clinical applications. There are two main types, an intestinal type and a diffuse type (7). Intestinal-type GC is characterised by tubular and glandular structures, and diffuse-type GC lacks cell-to-cell interactions (7). Diffuse GC is more likely an inherited GC caused by CDH1 germline mutations that encode E-cadherin protein and mutant p53, and intestinal-type GC is more frequently associated with claudin 6 overexpression (8). The diffuse type correlates with the genomic stable type, and the intestinal type is associated with the chromosomal instability type (5). The intestinal type has a closer relationship with *Helicobacter pylori* (*H.p.)* compared to the diffuse type, which is why the incidence of the intestinal type is diminished and the diffuse type GC is increased (9, 10). The diffuse type is prone to peritoneal metastasis, and the intestinal type has a higher risk of liver metastasis. Therefore, the recurrence patterns are also different between types (11). The prognosis is controversial based on the Lauren classification (8, 12). Early-stage gastric cancer (EGC), which comprises T1 tumours irrespective of lymph node metastasis (LNM), is a special type of cancer. EGC tumours are limited to the mucosa or submucosa, which have accounted for an increasing proportion with the rapid advocation of endoscopy, with a ratio of up to 60% (13). The proportion of early-onset GC is rare, and its features remain a knowledge gap for most clinical doctors and researchers. However, early-onset GC is more aggressive than traditional GC, which has prompted many researchers to focus on this research area (4, 14). Comparisons of the clinical features, prognosis and genomic features between intestinal-type and diffuse-type GC in early-onset early-stage GC (EEGC) were not performed. Therefore, we examined all the above work, and the results elucidate these differences.

Our manuscript included 581 EEGC cases based on the Lauren classification and 226 patients with EEGC from the First Affiliated Hospital of Nanchang University to analyse the differences in LNM, prognosis and genomic expression and found that intestinal-type EEGC had a higher rate of LNM and poorer prognosis. Intestinal-type EEGC and diffuse-type EEGC with LNM had different genomic expressions.

## Methods

### Extraction of Patient Data

All patient data were extracted from the SEER database and the First Hospital of Nanchang University. For extracting patient data from the SEER database using National Cancer Institute's SEER^*^Stat software (version 8.3.6), we designated the following inclusion criteria: (1) patients who were <50 years old and were diagnosed with T1 stage GC on histological examination; (2) patients with detailed records of survival information; (3) patients with concrete information of race, histological grade, examined lymph nodes (LNs), and tumour diameter; and (4) all patients who underwent surgery without chemotherapy before surgery. The following exclusion criteria were used: patients with unknown information about our included clinical features, such as tumour site and N stage; and patients who were not diagnosed with intestinal (M8140, M8211, M8010, and M8144) or diffuse type (M8145, M8490, and M8142) (9) disease. To extract patient data from our centre, we selected patients who were diagnosed from January 2011 to January 2020 to collect the clinical characteristics. The following inclusion criteria were used: (1) patients who were diagnosed with T1 stage GC by pathology and aged <50 years; (2) patients who did not receive preoperative adjuvant therapy; and (3) patients who had Lauren classification information. The exclusion criteria included (1) patients without records of T stage, N stage, and lymphatic invasion and (2) patients with severe diseases, such as cirrhosis, renal failure, and cardiac failure. All our patients were followed up *via* telephone or we-chat, and patients who missed their follow-up were excluded when we analysed the difference in prognosis. The concrete information of patients from the SEER database is listed in Table 1, and information on patients extracted from our centre is shown in Table 2.

**Table 1:** Basic characteristics of patients diagnosed with gastric cancer (GC) based on Lauren type in T1 stage from January 2010 to January 2015 in the SEER database.

**Variables**	**Total (%)**	**Intestinal type**	**Diffuse type**	***P* Value**
*n*	581	297	284	
**Age**				0.0019
20–29	35 (6.02%)	19 (6.40%)	16 (5.63%)	
30–39	135 (23.24%)	51 (17.17%)	84 (29.58%)	
40–45	411 (70.74%)	227 (76.43%)	184 (64.79%)	
**Race**				0.4387
White	393 (67.64%)	204 (68.69%)	189 (66.55%)	
Black	87 (14.97%)	47 (15.82%)	40 (14.08%)	
Other	101 (17.38%)	46 (15.49%)	55 (19.37%)	
**Sex**				<0.001
Male	313 (53.87%)	193 (64.98%)	120 (42.25%)	
Female	268 (46.13%)	104 (35.02%)	164 (57.75%)	
**Lymph node metastasis**				0.0001
No	418 (71.94%)	193 (64.98%)	225 (79.23%)	
Yes	163 (28.06%)	104 (35.02%)	59 (20.77%)	
**Tumour site**				<0.001
Cardia	140 (24.1%)	112 (37.71%)	28 (9.86%)	
Fundus	21 (3.61%)	9 (3.03%)	12 (4.22%)	
Body	69 (11.88%)	33 (11.11%)	36 (12.68%)	
Antrum	216 (37.18%)	88 (29.63%)	128 (45.07%)	
Overlappping/NOS	146 (25.13%)	66 (22.22%)	80 (28.17%)	
**T stage**				0.002
T1a	284 (48.88%)	123 (41.41%)	161 (56.69%)	
T1b	297 (51.12%)	174 (58.59%)	123 (43.31%)	
**Tumour size**				<0.001
≤3cm	17 (2.93%)	17 (5.72%)	0 (0.00%)	
>3cm	564 (92.07%)	280 (94.28%)	284 (100%)	
**Examined LNs**				0.0017
≤16	493 (84.85%)	266 (89.56%)	227 (79.93%)	
>16	88 (15.15%)	31 (10.44%)	57 (20.07%)	
**Cell differentiation**				<0.001
Well/moderately differentiated	125 (21.51%)	114 (38.38%)	11 (3.87%)	
Poorly differentiated/undifferentiated	456 (78.49%)	183 (61.62%)	273 (96.13%)	

**Table 2:** Basic information of patients with GC based on Lauren type in T1 stage from our hospital diagnosed from January 2011 to January 2019.

**Variables**	**Total**	**Intestinal type (IT)**	**Diffuse type (DT)**	**Mixed type (MT)**	***P* value (IT/DT)**
Total	226	101	88	37	
**Age (median)**	41	41	40	40	
**Sex**					0.017
Man	104 (46.02%)	57 (56.44%)	34 (38.64%)	13 (35.14%)	
Female	122 (53.98%)	44 (43.56%)	54 (61.36%)	24 (64.86%)	
**Paris type**					0.182
IIa/IIb	53 (23.45%)	20 (19.80%)	27 (30.68%)	6 (16.22%)	
IIc/III	173 (76.55%)	81 (80.20%)	61 (69.32%)	31 (83.78%)	
**Histopatholo-gic type**					-
Tubular adenocarcinoma	101 (44.69%)	101 (100%)	0 (0.00%)	0 (0.00%)	-
Signet ring cell carcinoma	26 (11.50%)	0 (0.00%)	26 (29.55%)	0 (0.00%)	-
Low adhesion carcinoma	62 (27.43%)	0 (0.00%)	62 (70.45%)	0 (0.00%)	-
Mixed carcinoma	37 (16.37%)	0 (0.00%)	0 (0.00%)	100 (100%)	
**Tumour site**					0.279
Fundus	3 (1.33%)	2 (1.98%)	1 (1.14%)	0 (0.00%)	
Body	40 (17.70%)	14 (13.86%)	20 (22.73%)	6 (16.22%)	
Antrum	183 (80.97%)	85 (84.16%)	67 (76.14%)	31 (83.78%)	
**Hp Positive**					0.745
No	81 (35.84%)	32 (31.68%)	31 (35.23%)	18 (48.65%)	
Yes	55 (24.34%)	25 (24.75%)	24 (27.27%)	6 (16.22%)	
Unknown	90 (39.82%)	44 (43.56%)	33 (37.50%)	13 (35.14%)	
**Cell differentiation**					<0.001
Poorly differentiated	121 (53.54%)	16 (15.84%)	76 (86.36%)	29 (78.38%)	
Moderately differentiated	92 (40.71%)	82 (81.19%)	5 (5.68%)	5 (13.51%)	
Well differentiated	10 (4.42%)	3 (2.97%)	7 (7.95%)	0 (0.00%)	
Unknown	3 (1.33%)	0 (0.00%)	0 (0.00%)	3 (8.11%)	
**Tumour size**					0.308
≤3 cm	133 (58.85%)	64 (63.37%)	49 (55.68%)	20 (54.05%)	
>3 cm	93 (41.15%)	37 (36.63%)	39 (44.32%)	17 (45.95%)	
**Examined_LNs**					0.62
<16	29 (12.83%)	15 (14.85%)	11 (12.5%)	3 (8.11%)	
≥16	197 (87.17%)	86 (85.15%)	77 (87.50%)	34 (91.89%)	
**Smoking**					0.109
No	166 (73.45%)	68 (67.33%)	69 (78.41%)	29 (78.38%)	
Yes	60 (26.55%)	33 (32.67%)	19 (21.59%)	8 (21.62%)	
**Drinking**					0.125
No	178 (78.76%)	75 (74.26%)	74 (84.09%)	29 (78.38%)	
Yes	48 (21.24%)	26 (25.74%)	14 (15.91%)	8 (21.62%)	
**Depth**					0.227
T1a	119 (52.65%)	49 (48.51%)	50 (56.82%)	20 (54.05%)	
T1b	107 (47.35%)	52 (51.49%)	38 (43.18%)	17 (45.95%)	
**N stage**					0.031
No	177 (78.32%)	73 (72.28%)	75 (85.23%)	29 (78.38%)	
Yes	49 (21.68%)	28 (27.72%)	13 (14.77%)	8 (21.62%)	
N1	31 (13.72%)	18 (17.82%)	9 (10.23%)	4 (10.81%)	
N2	12 (5.31%)	6 (5.94%)	3 (3.41%)	3 (8.11%)	
N3	6 (2.65%)	4 (3.96%)	1 (1.14%)	1 (2.70%)	
**Lymphatic invasion**				+	0.033
No	202 (89.38%)	88 (87.13%)	83 (94.32%)	30 (81.08%)	
Yes	24 (10.62%)	13 (12.87%)	3 (3.41%)	7 (18.92%)	
**Family history**					0.65
No	193 (85.40%)	85 (84.16%)	76 (86.36%)	32 (86.49%)	
Yes	33 (14.60%)	16 (15.84%)	12 (13.64%)	5 (13.51%)	

### Definition of Variables

The clinical features extracted from the SEER database included sex, race, primary tumour site, pathological grade, N stage, and examined LNs. Some additional features in our patients included *H.p*., smoking, drinking, invasive depth, family history, and lymphatic invasion. All patients were divided into intestinal-type and diffuse-type EEGC according to the definition. Sex was recorded as male and female. The race was separated into white, black, and other races. Primary sites included cardia, fundus, body, antrum, and overlapping tumours. The pathological grade was divided into four groups: well, moderately, poorly differentiated, and undifferentiated. N stage was recorded as negative (No) and positive (Yes). Tumour size was divided into ≤3 cm and >3 cm according to previous studies (15). Examined LNs were divided into <16 and ≥16 according to certain guidelines (16). Smoking, drinking, family history, and lymphatic invasion were listed as negative (No) and positive (Yes). The main observation features included LNM, overall survival (OS), and cancer-specific survival (CSS).

### Protein Mass Spectrometry Analysis

The entire process was completed by Shanghai Luming Biological Technology Co., Ltd. We collected paraffin tissue from 12 patients, including three pairs of intestinal-type EEGC with positive LNM or negative LNM and three pairs of diffuse-type EEGC, which underwent propensity score matching (PSM). All analyses were performed using a fusion mass spectrometer (Thermo, USA) equipped with an Easyspray source (Thermo, USA). All labelled samples were mixed with equal amounts of tandem mass tag (TMT) reagent. The samples were loaded by a capillary trap column (100 μm × 2 cm, RP-C18, Thermo Fisher) and separated using a capillary analytical column (15 cm × 75 μm, RP-C18, Thermo Fisher) on an EASY-nLCTM 1200 system (Thermo, USA). Full MS scans were acquired in the mass range of 350–1,500 m/z with a mass resolution of 1,20,000, and the AGC target value was set at 4e5. The 10 most intense peaks in MS were fragmented using higher-energy collisional dissociation with a collision energy of 38. MS/MS spectra were obtained with a resolution of 50,000 with an Automatic Gain Control (AGC) target of 5e4 and a maximum injection time of 86 ms. The fusion dynamic exclusion was set for 45 s and was run under positive mode. Spectronaut was used to perform a thorough search of all of the raw data against the sample protein database. A database search was performed with trypsin digestion specificity. Alkylation on cysteine was considered a fixed modification in the database search. The protein, peptide, and PSM false discovery rates (FDRs) were set to 0.01. For Data independent acquisition (DIA) data, the quantification FDR was also set to 0.05.

### Statistical Analysis

For basic statistical analysis, all extracted patients were divided into intestinal-type and diffuse-type EEGC according to Lauren classification, and the differences in the included clinical characteristics were compared using Pearson's chi-squared test. Univariate and multivariate logistic regression analyses were used to investigate the potential risk factors associated with LNM, and Cox regression analysis was used for the analysis of prognostic factors. All results are shown as odds ratios (ORs) and hazard ratios (HRs) with 95% CIs. For the imbalance between the two groups, we performed PSM to obtain new data for analysis, and the calliper value was set as 0.02. The effect was balanced when the *P* > 0.05. As described in our previous study (17), we completed the process in R software and determined the *P*-value using Pearson's chi-squared test. We examined the correlations with LNM between intestinal-type and diffuse-type EEGC using univariate logistic analysis and used univariate Cox regression analysis to assess whether intestinal-type and diffuse-type EEGC had different survival. All statistical analyses were performed using R software, and all associated packages of R software were obtained from the software program's website (https://cran.r-project.org/web/packages/). The student's *t*-test was used for continuous variables with a Gaussian distribution, and the non-parametric Kruskal–Wallis rank-sum test was used for non-normally distributed continuous variables or ordinal categorical variables. The chi-squared test was performed using SPSS (version 24.0). The results were statistically significant when the *P* < 0.05.

## Results

### Basic Information of Patients From the SEER Database and Our Hospital

According to the flowchart in Supplementary Figure S1, we included 581 patients with EEGC, 297 intestinal-type and 284 diffuse-type GC patients. The flowchart in Supplementary Figure S2 shows that we enrolled 226 patients with EEGC, which included 101 intestinal-type patients, 88 diffuse-type patients, and 37 mixed-type patients who were diagnosed from January 2011 to January 2020. The basic information of patients from the SEER database is listed in Table 1, and information on patients from our hospital is shown in Table 2. Patients with diffuse-type were younger and more likely to be female than patients with intestinal-type (*P* < 0.05) (Table 1). Diffuse-type GC was superior to be poorly differentiated GC and had more examined LNs (*P* < 0.05). Diffuse-type GC was superior to poorly differentiated GC and had more examined LNs (*P* < 0.05). However, we found that intestinal-type EEGC was more likely positive for LNM (35.02 vs. 20.77%, *P* = 0.0001) and invasive (T1b, 58.59 vs. 43.31%, *P* = 0.002). Our patients with diffuse-type GC were more likely to be female and have poor differentiation (*P* < 0.05) (Table 2), and patients with intestinal-type GC tended to have positive LNM (27.72 vs. 14.77%, *P* = 0.031) and lymphatic invasion (12.87 vs. 3.41%, *P* = 0.033). We also found that patients with mixed-type GC had high rates of LNM (21.62%) and lymphatic invasion (18.92%). However, we did not compare mixed-type GC with the other two types due to the limited sample of the type.

### Comparison of LNM and Identification of Risk Factors for LNM in Patients With Intestinal-Type and Diffuse-Type EEGC

To investigate the risk factors for LNM, we performed univariate logistic regression analysis. The SEER data (Table 3) revealed that advanced T stage (T1b vs. T1a, OR, 1.98, *P* = 0.014) and larger tumour size (>3 cm vs. <3 cm, OR, 3.62, *P* < 0.001) were risk factors. We found that diffuse-type GC was less likely to cause LNM (OR, 0.375; 95% CI, 0.24–0.586, *P* < 0.001). For our patients (Table 4), women tended to have positive LNM (OR, 4.308; 95% CI, 1.701–10.9, *P* = 0.002), larger tumour size, T1b stage, and positive lymphatic invasion (OR, 24.285; 95% CI, 5.878–100.46, *P* < 0.001). We found that diffuse type was a protective factor for LNM (OR, 0.358; 95% CI, 0.147–0.873). To adjust the confounding factors between the diffuse type and intestinal type, we performed PSM by matching 102 intestinal-type with 102 diffuse-type GC to determine whether Lauren type was associated with LNM. We found that the intestinal type was frequently associated with positive LNM (35.02 vs. 20.77%, *P* = 0.001) (Supplementary Table S1).

**Table 3:** Univariate and multivariate logistic regression model for exploring the potential risk factors for lymph node metastasis (LNM) in patients from the SEER database.

**Variables**	**Univariate analysis**	***P* Value**	**Multivariate analysis**	***P* Value**
**Age**				
20–29	Reference	-		
30–39	1.25 (0.553–2.827)	0.592		
40–45	0.891 (0.414–1.916)	0.768		
**Race**				
White	Reference	-		
Black	0.659 (0.379–1.145)	0.139		
Other	0.861 (0.527–1.406)	0.549		
**Sex**				
Male	Reference	-	Reference	-
Female	0.588 (0.406–0.853)	0.005	0.749 (0.502–1.118)	0.749
**Lauren type**				
Intestinal type	Reference	-	Reference	-
Diffuse type	0.487 (0.335–0.707)	<0.001	0.375 (0.240–0.586)	<0.001
**Tumour site**		0.001		0.157
Cardia	Reference	-	Reference	-
Fundus	1.125 (0.445–2.846)	0.084	1.332 (0.501–3.547)	0.566
Body	0.57 (0.304–1.067)	0.079	0.695 (0.359–1.345)	0.28
Anturm	0.398 (0.247–0.639)	<0.001	0.6081 (0.311–1.053)	0.013
Overlappping/NOS	0.495 (0.297–0.825)	0.007	0.354 (0.24–0.586)	0.076
**T stage**				
T1a	Reference	-	Reference	-
T1b	1.698 (1.212–2.946)	0.034	1.98 (1.352–3.146)	0.014
**Tumour size**				
≤3 cm	Reference	-	Reference	-
>3 cm	1.711 (1.072–2.733)	0.024	3.62 (1.957–5.633)	<0.001
**Examined LNs**				
≤16	Reference	-		
>16	0.688 (0.409–0.894)	0.012		
**Cell differentiation**				
Well/moderately differentiated	Reference	-		
Poorly differentiated/undifferentiated	-	-		

**Table 4:** Univariate and multivariate logistic regression model for exploring the potential risk factors for LNM in patients from our hospital.

**Variables**	**Univariate analysis**	***P* Value**	**Multivariate analysis**	***P* Value**
**Age**				
20–39 years	Reference	-		
40–45 years	1.473 (0.722–3.006)	0.287		
**Sex**				
Man	Reference	-	Reference	-
Female	3.209 (1.496–6.88)	0.003	4.308 (1.702–10.9)	0.002
**Paris type**				
IIa/IIb	Reference	-		
IIc/III	1.251 (0.528–2.966)	0.611		
**Hp Positive**				
No	Reference	-		
Yes	0.877 (0.399–1.924)	0.743		
Unknown	0.847 (0.373–1.921)	0.691		
**Cell differentiation**				
Poorly differentiated	Reference	-		
Moderately/well differentiated	1.001 (0.488–2.055)	0.997		
Unknown	-	-		
**Tumour size**				
≤3 cm	Reference	-	Reference	-
>3 cm	2.29 (1.135–4.623)	0.021	2.423 (1.038–5.653)	0.041
**Examined LNs**				
<16	Reference	-		
≥16	1.036 (0.359–2.929)	0.962		
**Smoking**				
No	Reference	-		
Yes	1.063 (0.808–1.561)	0.052		
**Drinking**				
No	Reference	-		
Yes	0.391 (0.13–1.177)	0.095		
**Depth**				
T1a	Reference	-	Reference	-
T1b	2.275 (1.114–4.645)	0.024	2.259 (0.965–5.289)	0.046
**Lauren type**				
Intestinal type	Reference	-	Reference	-
Diffuse type	0.452 (0.217–0.94)	0.034	0.358 (0.147–0.873)	0.024
**Lymphatic invasion**				
No	Reference	-	Reference	-
Yes	25.062 (6.743–93.15)	<0.001	24.285 (5.878–100.46)	<0.001
**Family history**				
No	Reference	-		
Yes	1.552 (0.628–3.834)	0.341		

### Comparison of Survival and Identification of Risk Factors for Survival Between Patients With Intestinal-Type EEGC and Patients With Diffuse-Type EEGC

**Figure 1:**
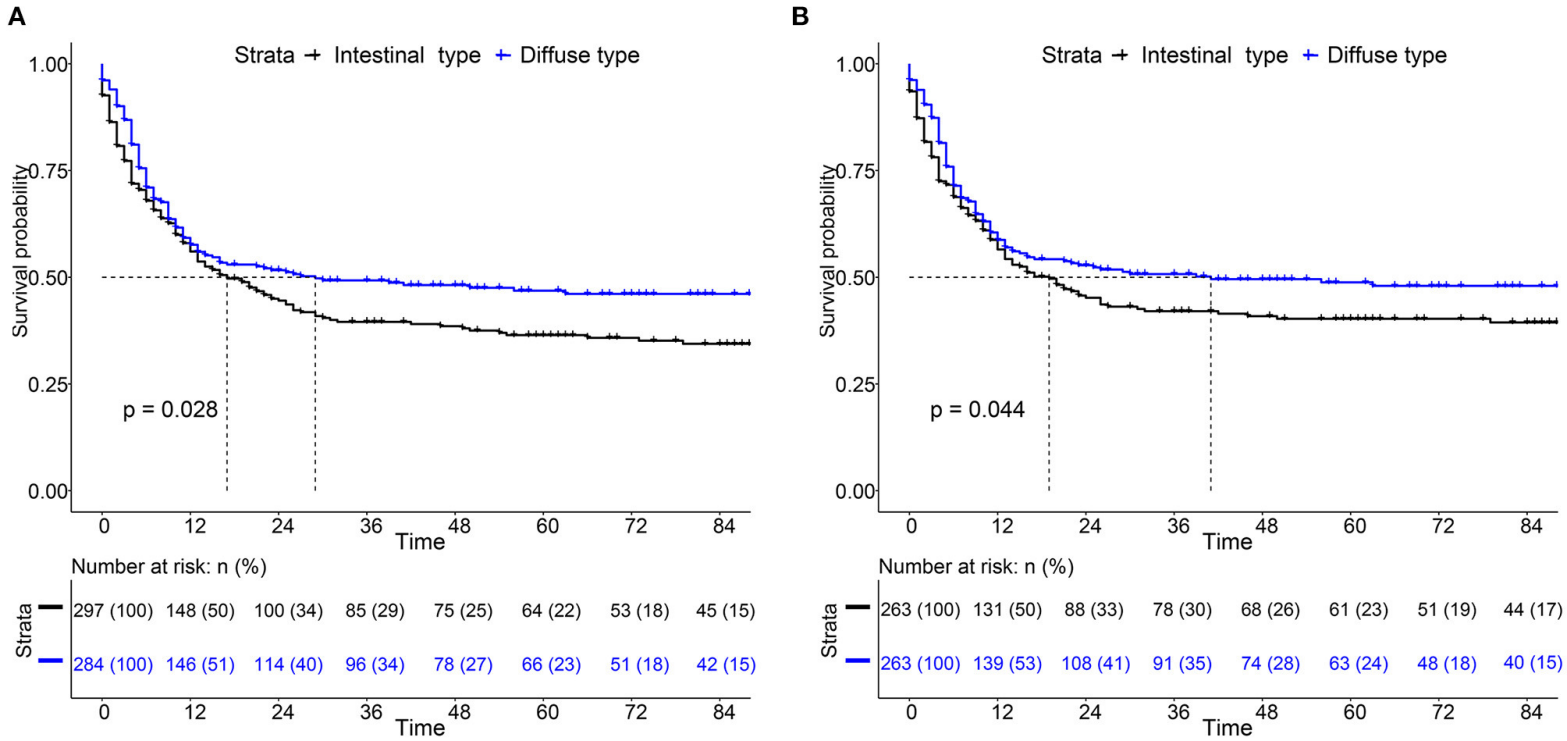
Comparison of survival between intestinal type and diffuse type early-onset early-stage GC (EEGC). **(A,B)** K-M survival curve of overall survival (OS) and cancer-specific survival (CSS) between the two groups, respectively.

**Figure 2:**
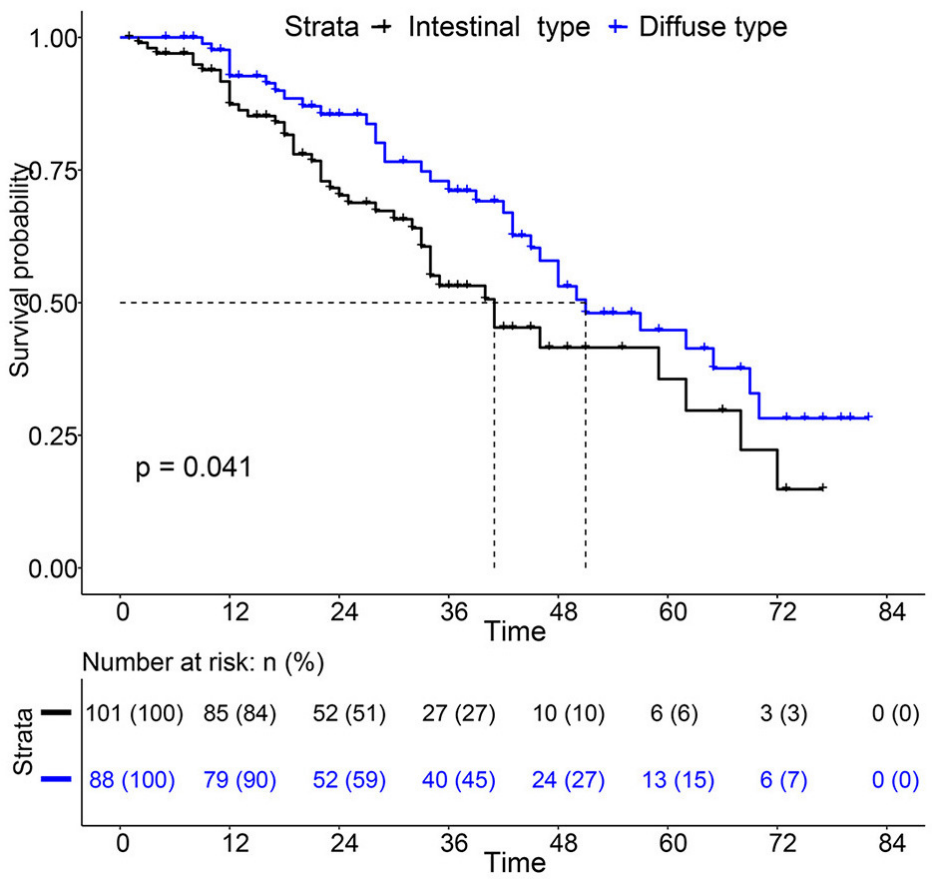
K-M survival curve between Intestinal type and diffuse type EEGC was performed with data from our hospital.

**Figure 3:**
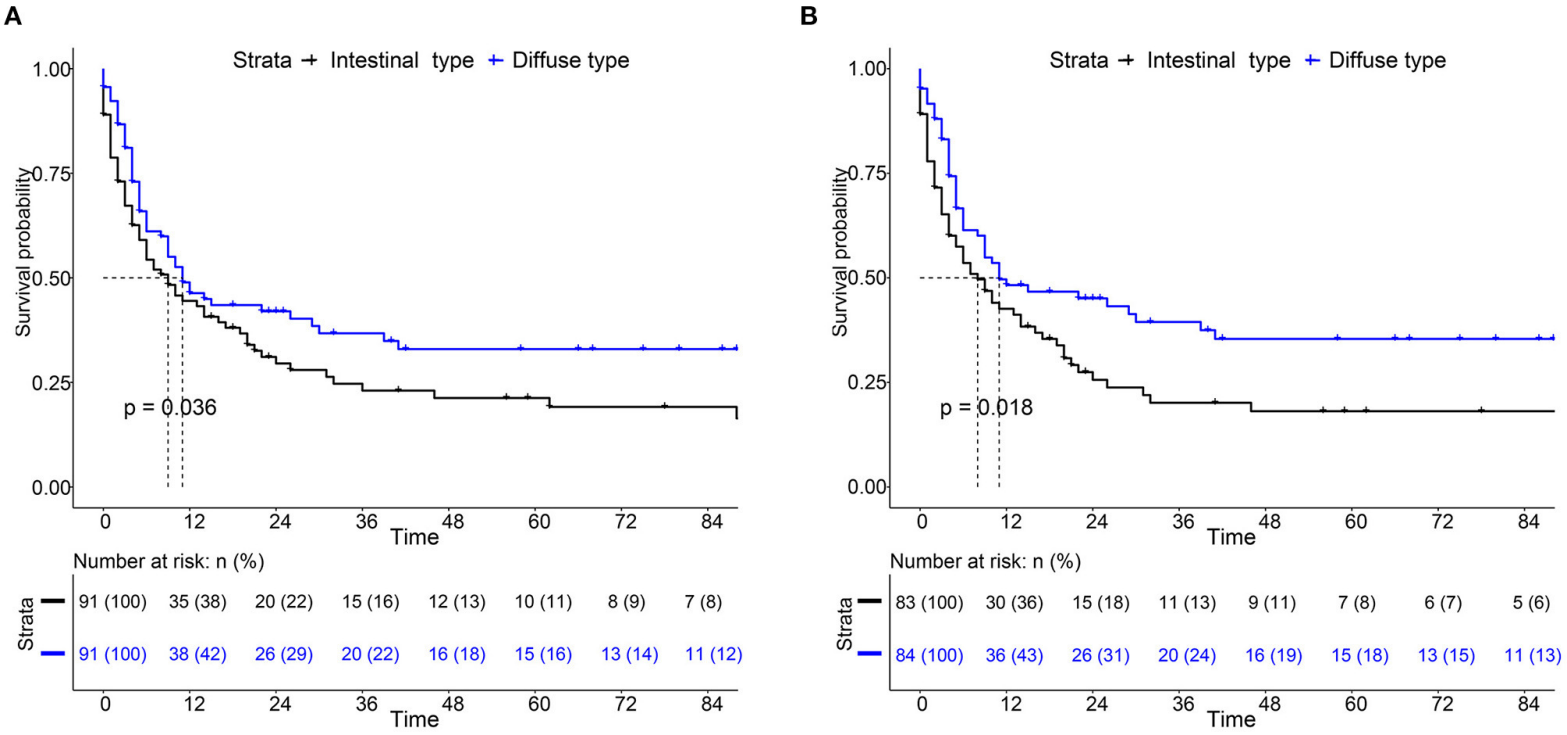
K-M survival curve of OS **(A)** and CSS **(B)** between intestinal type and diffuse type EEGC after propensity score matching (PSM) by SEER data.

**Figure 4:**
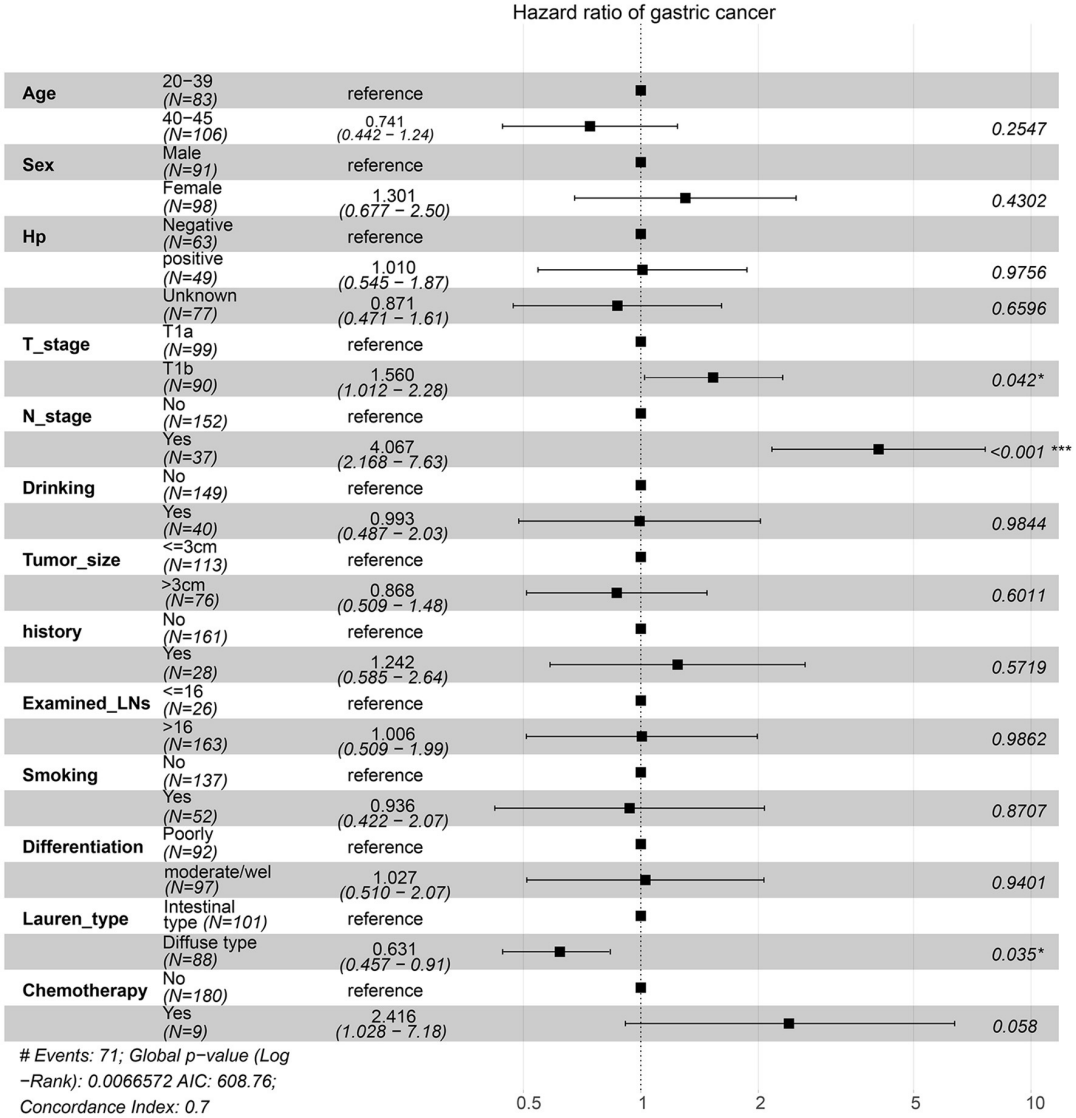
Risk factors associated with survival were identified by multivariate Cox regression analysis using the information of patients from our centre.

For the survival between the two types, we first plotted the Kaplan-Meier curve (K-M curve) survival curve of the SEER data and our data. As shown in Figure 1, patients with diffuse-type had a better prognosis of OS and CSS (*P* < 0.05). Our own data also revealed that diffuse type was a protective factor for prognosis (*P* < 0.05) (Figure 2). Because some clinical features were not balanced in the SEER data, we performed PSM to eliminate confounding factors that correlated with survival (Supplementary Table S2) and found that patients with diffuse-type disease had a better prognosis (Figure 3). To explore related hazard factors, we performed univariate and multivariate Cox analyses, which suggested that positive LNM, T1b stage, and intestinal type were independent risk factors for patients with EEGC from the SEER data and our centre (Table 5 and Figure 4).

**Table 5:** Univariate and multivariate cox regression model for exploring the potential risk factors for patients' overall survival (OS) from SEER database.

**Variables**	**Univariate analysis**	***P* Value**	**Multivariate analysis**	***P* Value**
**Age**				
20–29	Reference	-		
30–39	1.441 (0.816–2.546)	0.208		
40–45	1.184 (0.69–2.033)	0.54		
**Race**				
White	Reference	-		
Black	1.248 (0.929–1.675)	0.141		
Other	0.722 (0.517–1.09)	0.056		
**Sex**				
Male	Reference	-		
Female	0.893 (0.714–1.118)	0.324		
**Lymph node metastasis**				
No	Reference	-	Reference	-
Yes	1.949 (1.546–2.456)	<0.001	2.118 (1.522–2.946)	<0.001
**Tumour site**				
Cardia	Reference	-		
Fundus	1.4 (0.796–2.462)	0.242		
Body	0.924 (0.632–1.352)	0.685		
Anturm	0.611 (0.453–0.825)	0.001		
Overlappping/NOS	1.163 (0.864–1.564)	0.319		
**T stage**				
T1a	Reference	-	Reference	-
T1b	2.153 (1.56–3.262)	0.022	2.068 (1.468–3.469)	0.035
**Tumour size**				
≤3 cm	Reference	-	Reference	-
>3 cm	1.667 (1.24–2.241)	0.001	1.708 (1.341–3.41)	<0.001
**Lauren type**				
Intestinal type	Reference	-	Reference	-
Diffuse type	0.792 (0.633–0.991)	0.041	0.644 (0.498–0.831)	0.001
**Examined LNs**				
≤16	Reference	-	Reference	-
>16	0.807 (0.624–0.968)	0.021	0.901 (0.821–1.238)	0.051
**Cell differentiation**				
Well/moderately differentiated	-	-		
Poorly differentiated/undifferentiated	-	-		

### Mass Spectrometry Analysis Found Several Special Proteins Associated With LNM Between Intestinal-Type and Diffuse-Type EEGC

**Figure 5:**
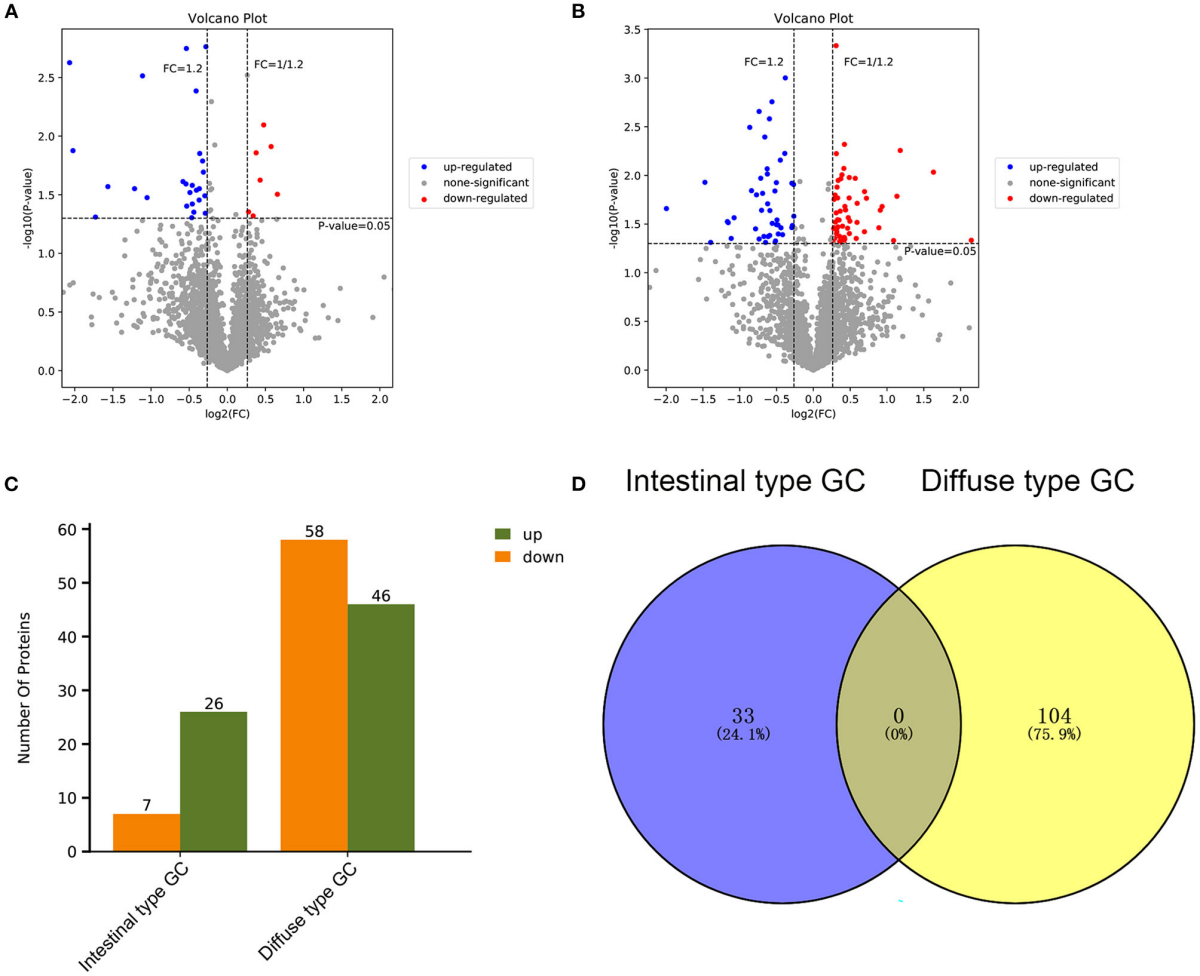
Differentially expressed genes (DEGs) of patients with positive LNM were investigated by performing protein mass spectrometry analysis compared to patients with negative lymph node metastasis (LNM) between intestinal type EEGC and diffuse type EEGC. **(A,B)** The DEGs were shown by volcano plot between intestinal type EEGC **(A)** and diffuse type EEGC **(B)**. **(C)** The histogram was performed to show the total DEGs between the two types. **(D)** Venn plot was performed to assess whether there were common DEGs between the two types.

**Figure 6:**
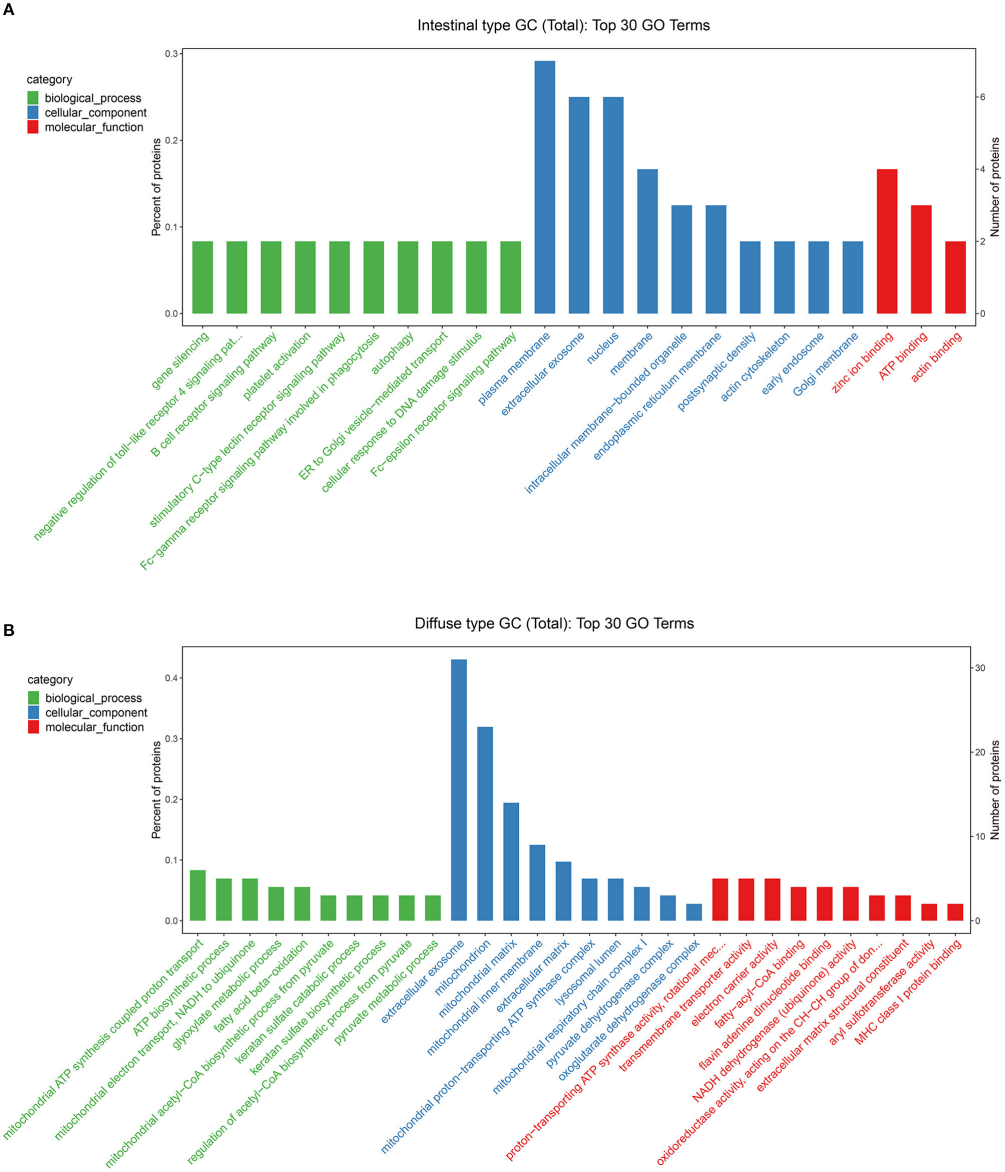
Gene Ontology (GO) analysis was performed to evaluate the biological process, cellular component, and molecular function of DEGs between intestinal type EEGC **(A)** and diffuse type EEGC **(B)**.

**Figure 7:**
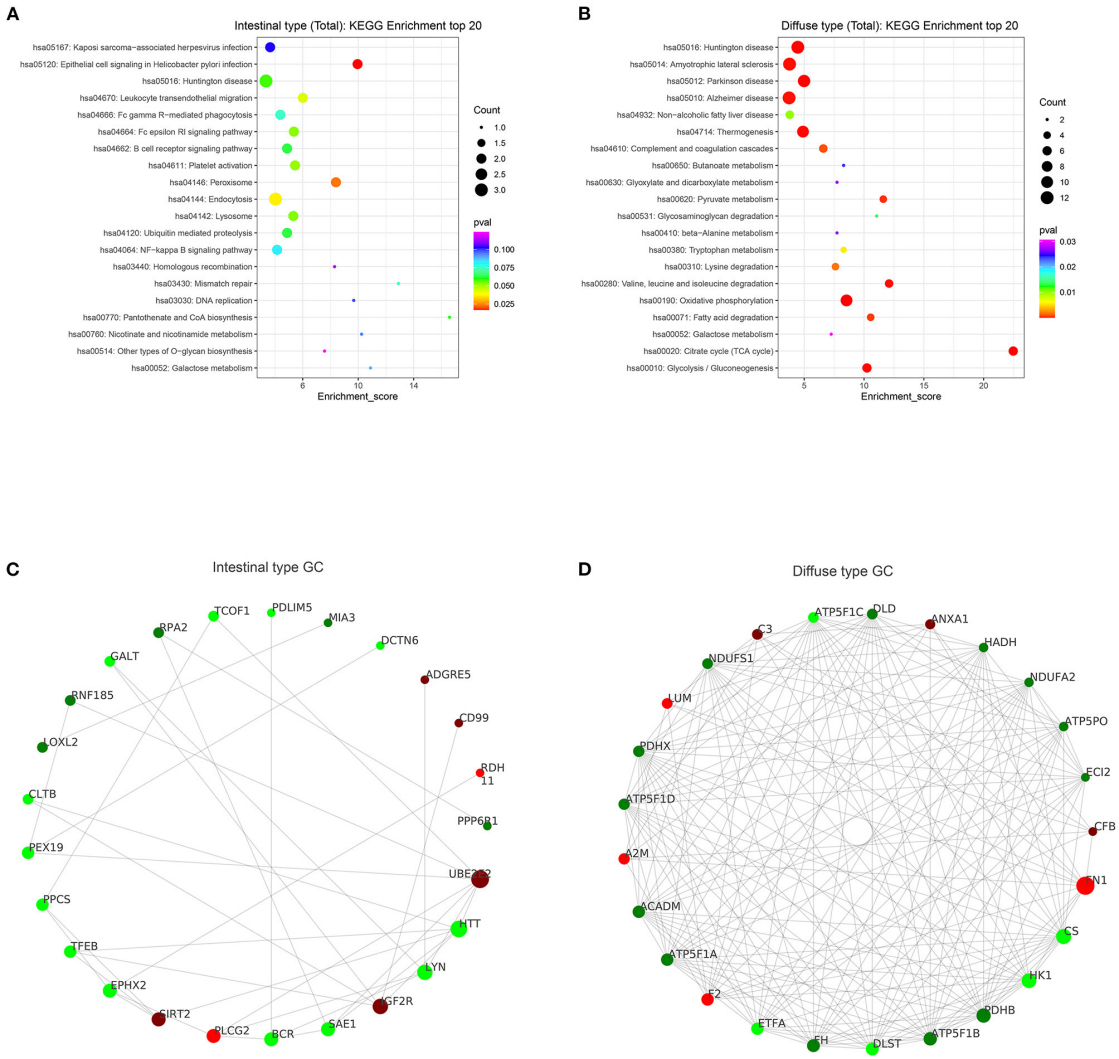
KEGG enrichment and protein interaction were performed according to DEGs. **(A,B)** results of KEGG enrichment between intestinal type EEGC **(A)** and diffuse type EEGC **(B)**. **(C,D)** results of protein-protein interaction between intestinal type EEGC **(C)** and diffuse type EEGC **(D)**.

Based on the results of our analysis, we considered that intestinal-type EEGC was prone to LNM, but the mechanism was not clear. Therefore, to explore the potential causes, we performed mass spectrometry analysis by extracting proteins from EEGC tissue blocked in wax. Regardless of intestinal-type or diffuse-type EEGC, we matched three patients who had positive LNM with three patients without LNM to exclude the influence of confounding factors. As shown in Figure 5, we found upregulated and downregulated genes in the two types of EEGC (Figures 5A,B), including 26 upregulated and seven downregulated genes in intestinal-type EEGC with positive LNM, and 46 upregulated and 58 downregulated genes in diffuse-type EEGC with LNM (Figure 5C). However, there were no common genes between these groups (Figure 5D). Detailed information on differentially expressed genes (DEGs) is shown in heat plots in Supplementary Figure S3 (intestinal type) and Supplementary Figure S4 (diffuse type), and the corresponding annotations are shown in Supplementary Tables S3,S4. For the intestinal type, the seven downregulated genes were UBE2E2, RDH11, IGF2R, ADDGRE5, PLCG2, SIRT2, and CD99, and the upregulated genes included DCTN6, SAE1, RNF185, and PEX19 (Supplementary Table S3). The upregulated genes in the diffuse type included FN1, SERPINB6, MUC6, and LAMB3, and the downregulated genes included HIP1R, PANK4, NDUFA2, and ITGA9 (Supplementary Table S4). Further GO analysis showed that DEGs in intestinal-type GC were involved in several biological processes, such as autophagy, DNA damage, and receptor signalling pathways, which were primarily plasma membrane, extracellular exosome, and other cellular components (Figure 6A). Unlike the intestinal type, the diffuse-type DEGs were primarily involved in mitochondrial ATP synthesis and glyoxylate metabolic processes, which consisted of extracellular exosomes, mitochondria, and other cellular components (Figure 6B). Kyoto Encyclopedia of Genes and Genomes (KEGG) pathway analysis found that DEGs in intestinal-type GC functioned in epithelial cell signalling in *H.p*. infection, followed by peroxisome and endocytosis (Figure 7A). We found that DEGs in the diffuse type were involved in the citrate cycle (TCA cycle), glycolysis/gluconeogenesis, and oxidative phosphorylation (Figure 7B). We constructed a protein-protein interaction network using Cytoscape software (Figures 7C,D). As shown in Figure 7C, we found that the UBE2E2, HTT, LYN, SIRT2, IGF2R, and SAE1 proteins had many interacting proteins. Unlike the intestinal type, the network was more complicated among DEGs (Figure 7D). FN1, ATP5F, and PDHX almost interacted with other DEGs.

## Discussion

Nowadays, few studies focused on early-onset GC in the T1 stage. Our study included 581 patients with EEGC from the SEER database and 226 patients with EEGC from our own centre. Both data revealed that intestinal type, T1b stage, and tumour size (>3 cm) were risk factors for LNM and intestinal type, T1b stage and positive LNM were hazard factors for survival. The DEGs in the intestinal type functioned as epithelial cell signalling in *H.p.*, and DEGs in the diffuse type functioned in the TCA cycle and oxidative phosphorylation. To our knowledge, this report is the first study to elucidate the differences in clinical features and genomic expression between intestinal- and diffuse-type EEGC.

For the two histological subtypes based on the Lauren type, some studies described the different clinical characteristics. Intestinal-type GC was superior to older patients compared to diffuse-type GC, which is also reported by other studies (9, 18). Some studies found that the incidence of the intestinal type increased faster than the diffuse type, and it was more frequent in male patients (11, 19). Therefore, this result may explain why the ratio of male patients with intestinal-type GC was larger than diffuse-type GC. Consistent with our results that the number of examined LNs was different between intestinal type and diffuse type, Arco et al. also found that the number of examined LNs varied in the Lauren type (20). Other differences in T stage, tumour site, and tumour site between intestinal-type and diffuse-type EEGC were reported in other studies (21). Unlike our previous knowledge that diffuse-type advanced GC was a risk factor for LNM (22–25), we found that intestinal type in EEGC was a risk factor associated with LNM (26). Other studies suggested a comparable rate of LNM between diffuse-type and intestinal-type GC for early-stage GC (21). Several reasons may explain these contradictory results. First, early-onset GC was not like conventional GC. Regardless of genomic mutation or inductive factors, they are distinct diseases (11, 27). Early-onset GC exhibited an E-cadherin-high, cyclooxygenase-2 (COX-2)-low, trefoil factor 1 (TFF1)-expressing phenotype, and COX-2 overexpression and loss of TFF1 were found in conventional cancers (28, 29). Second, diffuse-type and intestinal-type early-onset GC were also different, which may result in different clinical manifestations. For example, diffuse-type GC had a more germline mutation phenotype in CDH1, and intestinal-type GC was more likely due to *H.p*. infection (29, 30). Other than the Lauren type associated with LNM, in line with our results, some studies also reported submucosal tumour invasion, lympho-vascular invasion, and high-grade tumour differentiation as risk factors for LNM (31, 32).

To determine the association between Lauren classification and survival, we compared the disparity of survival in patients with diffuse-type and intestinal-type EEGC. To the best of our knowledge, this report is the first study to compare their survival. For conventional GC, several studies reported that patients with intestinal-type had better survival than patients with diffuse-type (18, 33), but some studies suggested that diffuse-type early GC was similar or better than intestinal-type GC (21, 34, 35). Our results of the K-M survival curve and Cox regression analysis before or after PSM showed that patients with intestinal-type EEGC had a worse prognosis, which sufficiently supported that intestinal-type EEGC was a risk factor for survival.

For the proteogenomic characterisation of early-onset GC, most studies focused on the diffuse type (36). Diffuse-type GC and intestinal-type GC are heterogeneous diseases with different molecular subtypes. For example, intestinal-type GC had a higher rate of microsatellite-unstable tumours, and diffuse-type GC had more mesenchymal-like types. Different genomic expression levels predicted different prognoses and recurrences (36, 37). Mun et al. performed an integrated proteogenomic analysis in young diffuse-type GC and found that many DEGs, such as MUC5B, CDH1, and LAMC1, were associated with phosphorylation or glycosylation, which may activate somatic mutations (38). Consistent with these findings, our results found that DEGs, such as LAMB and MUC6, were involved in the TCA cycle and oxidative phosphorylation. These DEGs were associated with metabolism and tumour invasion. Our study found that DEGs in the intestinal type were highly associated with *H.p*. infection, such as SAE1, RDH11, and RNF185. As indicated in epidemiological studies, *H.p*. infection was an important factor for intestinal-type GC (39). Previous studies showed that DEGs in intestinal-type GC were involved in *H.p*.-induced inflammation, and nuclear factor (NF)-κB signalling and proteins related to nitric oxide synthase or DNA damage promote the development and invasion of intestinal-type GC (39–41). Therefore, our results were similar to a previous study, which suggests that our results are reliable. However, our results were only based on three pairs of GC waxed tissue with negative or positive LNM, which may cause a limited number of DEGs and may be less convincing. To some extent, performing PSM reduces confounding factors and enhances reliability. Therefore, our DEGs between EEGC with negative LNM and EEGC with positive LNM may explain why intestinal-type EEGC had a higher rate of LNM than diffuse-type EEGC.

## Conclusion

In conclusion, our study was the first report to demonstrate the clinical characteristics between intestinal-type EEGC and diffuse-type EEGC and found that the intestinal type was a risk factor for LNM and survival. The oncogenic expression promoting the occurrence of LNM for the intestinal type was different from the diffuse type, which suggests that the mechanisms of LNM between the intestinal type and the diffuse type are unique. These findings may implicate that clinicians could recommend shorter follow-up intervals or maybe adjuvant therapy to intestinal-type EEGC than diffuse-type EEGC because of the higher rate of LNM and poorer rate of survival, however, the interesting findings would deserve being demonstrated by a larger-population study.

## Data Availability Statement

The original contributions presented in the study are included in the article/Supplementary Material, further inquiries can be directed to the corresponding author/s.

## Ethics Statement

The studies involving human participants were reviewed and approved by First Affiliated Hospital of Nanchang University. The patients/participants provided their written informed consent to participate in this study.

## Author Contributions

S-HC and C-TT: experiment performing, data analysis, and manuscript writing. S-HC: project development. All authors contributed to the article and approved the submitted version.

## Funding

Youth cultivation project of the Institute of First Affiliated Hospital of Nanchang University (YFYPY202023).

## Conflict of Interest

The authors declare that the research was conducted in the absence of any commercial or financial relationships that could be construed as a potential conflict of interest.

## Publisher's Note

All claims expressed in this article are solely those of the authors and do not necessarily represent those of their affiliated organizations, or those of the publisher, the editors and the reviewers. Any product that may be evaluated in this article, or claim that may be made by its manufacturer, is not guaranteed or endorsed by the publisher.

## Abbreviations

EEGC: early-onset early-stage GC
PSM: propensity score matching
DEGs: differentially expressed genes
LNM: lymph node metastasis
H.p: Helicobacter pylori
SEER: Surveillance, Epidemiology, and End Results Program
OS: overall survival
CSS: cancer-specific survival
OR: odd ratio.
