# Supplementary figures and images for: Higher Lymph Node Metastasis Rate and Poorer Prognosis of Intestinal-Type Gastric Cancer Compared to Diffuse-Type Gastric Cancer in Early-Onset Early-Stage Gastric Cancer: A Retrospective Study
Source: Front Med (Lausanne). 2021 Dec 23;8:758977. doi: 10.3389/fmed.2021.758977 (PMC8732774; doi:10.3389/fmed.2021.758977)

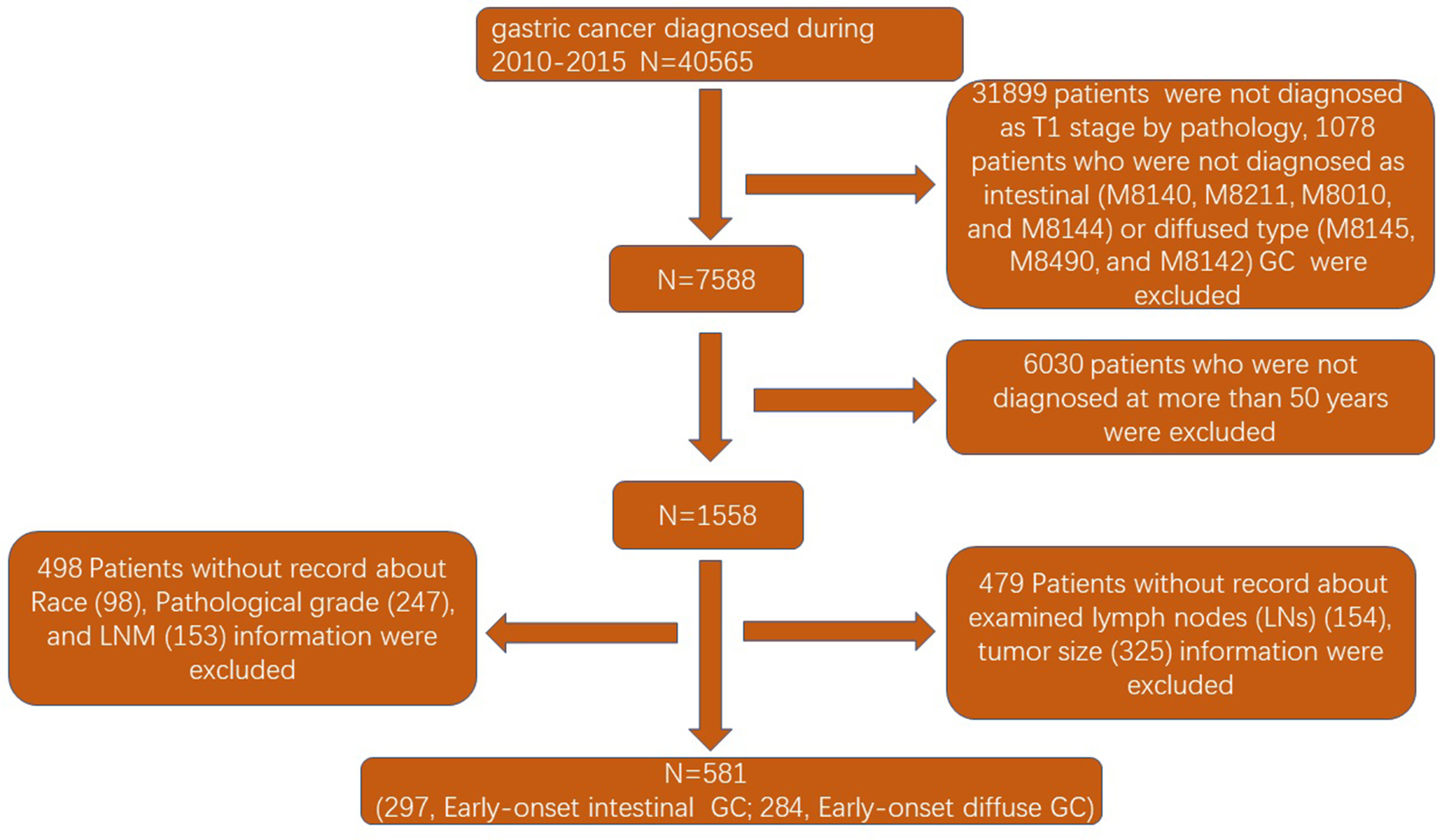

Supplement: Supplementary file 1 [file Image_1.JPEG]

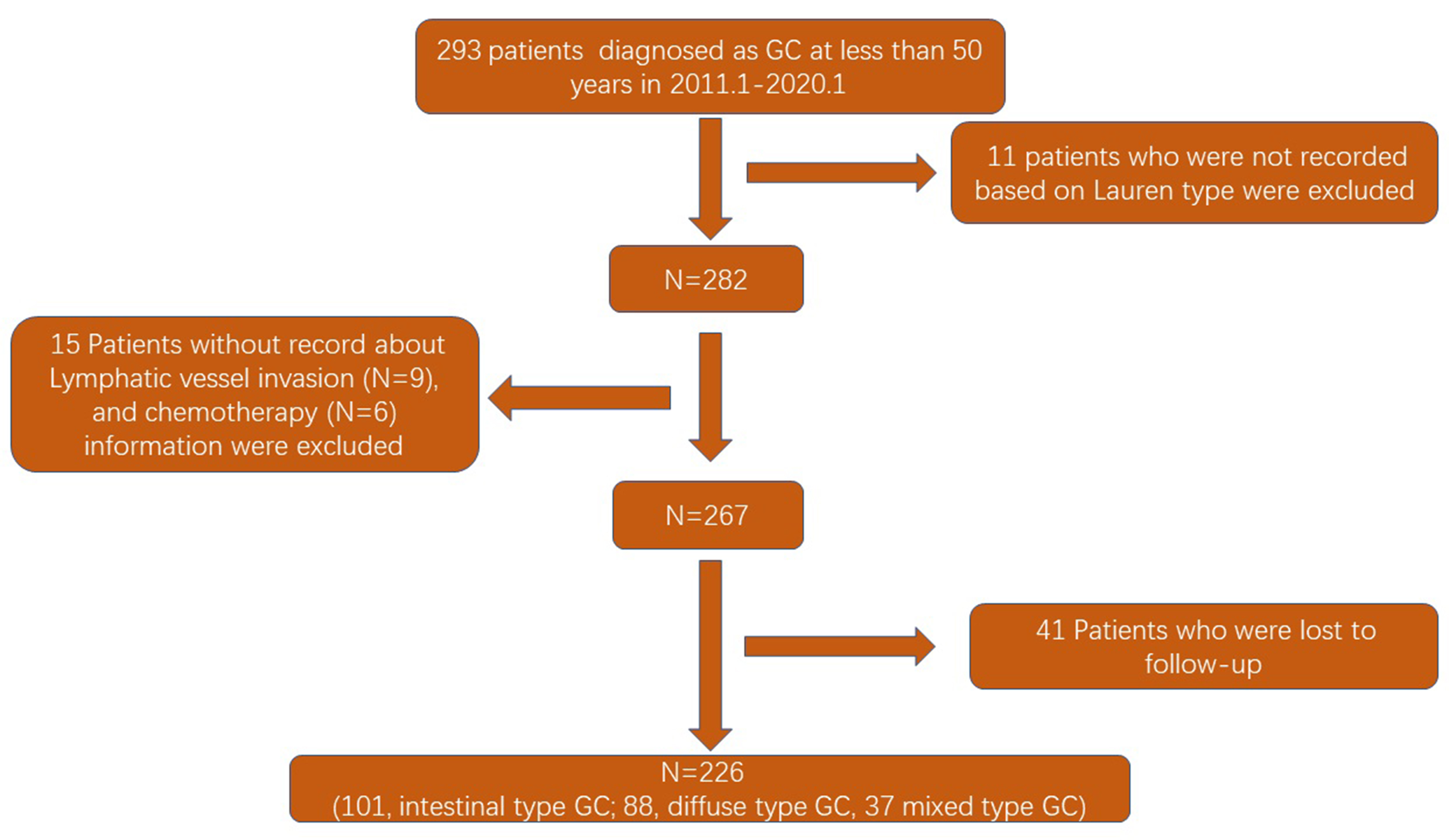

Supplement: Supplementary file 2 [file Image_2.JPEG]

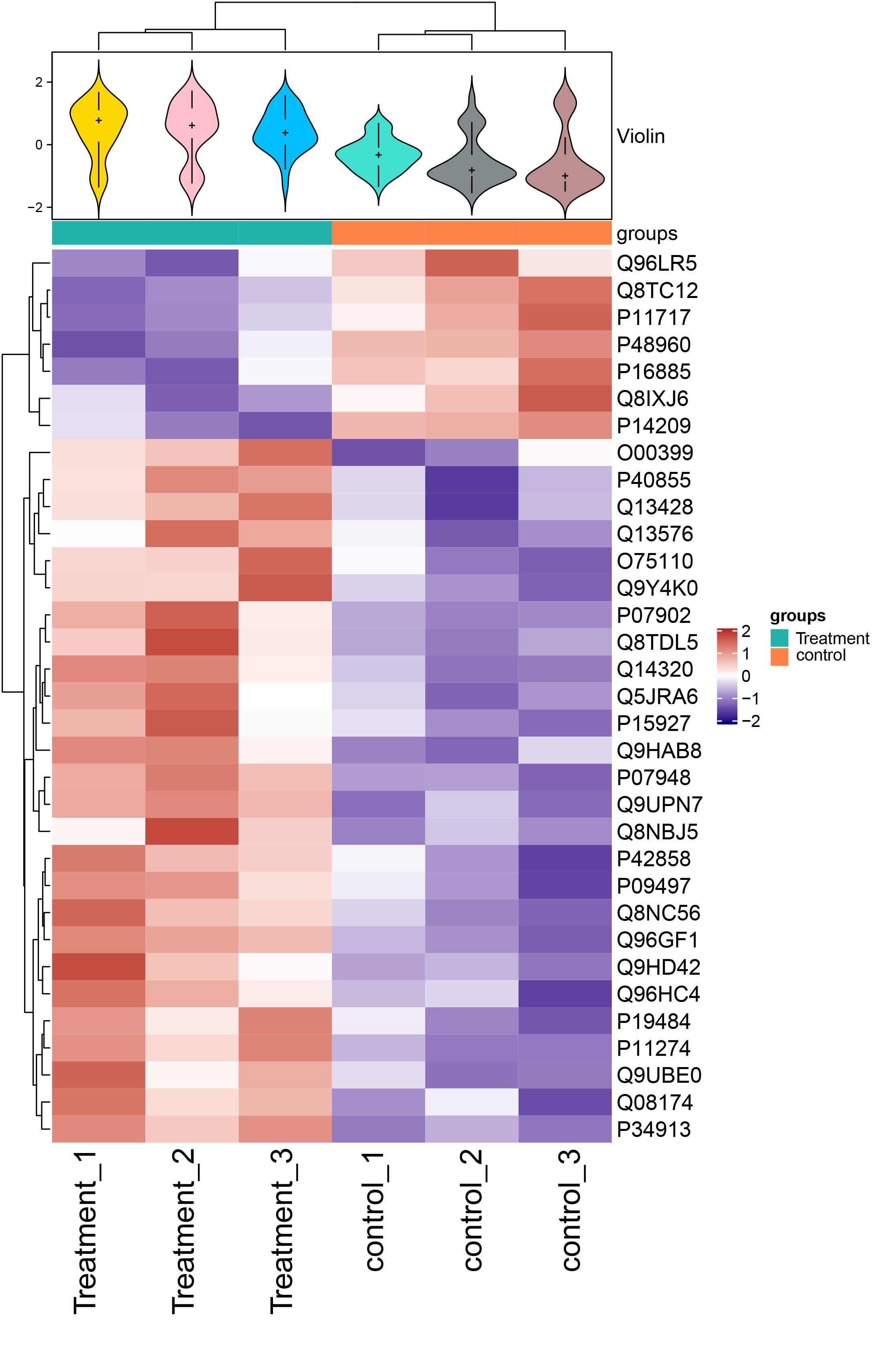

Supplement: Supplementary file 3 [file Image_3.JPEG]

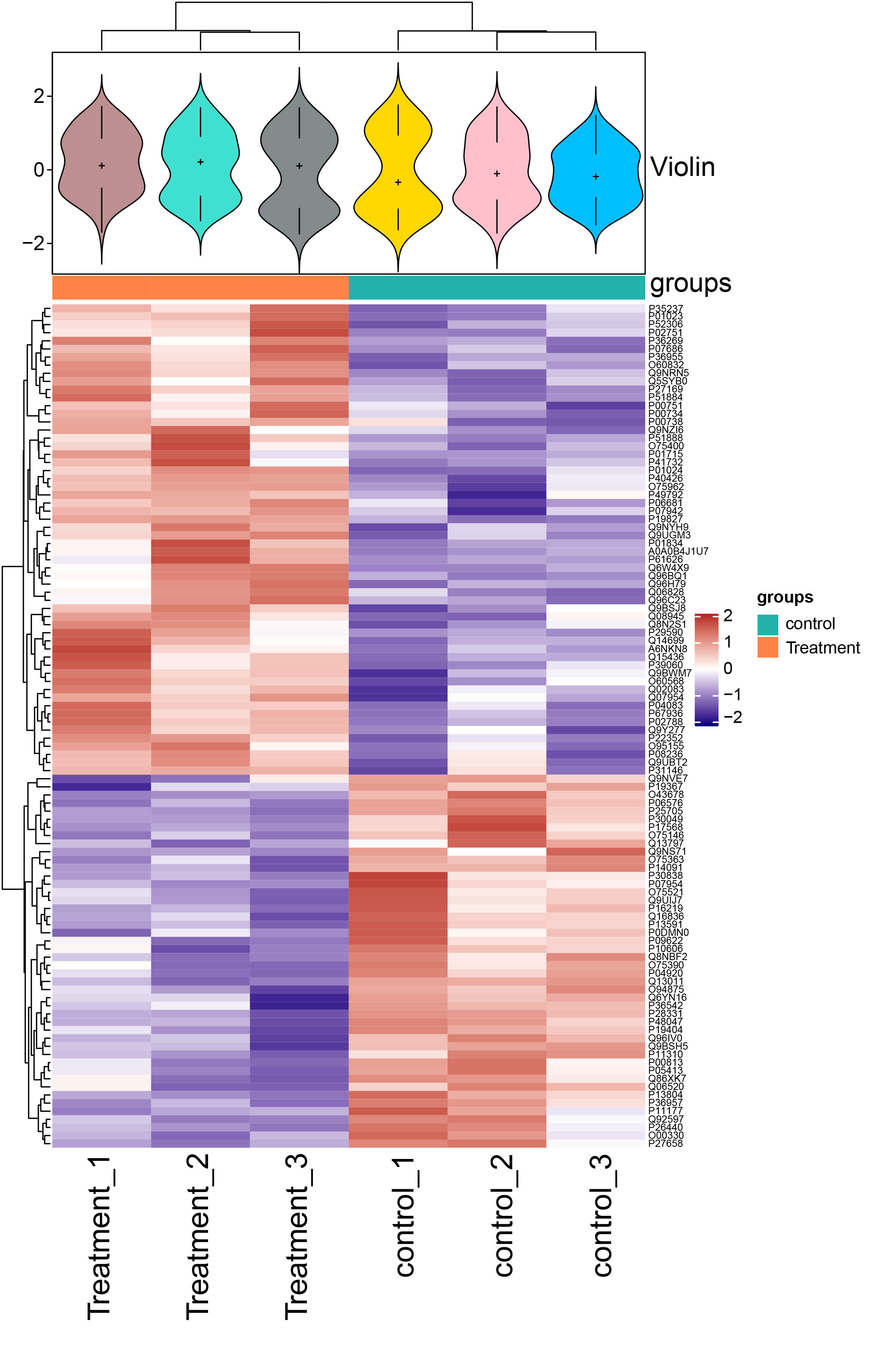

Supplement: Supplementary file 4 [file Image_4.JPEG]
