# Supplementary material for: Higher Lymph Node Metastasis Rate and Poorer Prognosis of Intestinal-Type Gastric Cancer Compared to Diffuse-Type Gastric Cancer in Early-Onset Early-Stage Gastric Cancer: A Retrospective Study
Source: Front Med (Lausanne). 2021 Dec 23;8:758977. doi: 10.3389/fmed.2021.758977 (PMC8732774; doi:10.3389/fmed.2021.758977)
Supplement: Supplementary file 5 [file Data_Sheet_1.docx]

**Supplementary figure 1:** The flow chart of extracting information of patients from SEER database.

**Supplementary figure 2:** The flow chart of extracting information of patients from the First affiliated hospital of Nanchang university.

**Supplementary figure 3:** The heat plot was performed to show DEGs in intestinal type EEGC which were identified by protein mass spectrometry analysis. Treatment group was patients with positive LNM, while control group was patients with negative LNM.

**Supplementary figure 4:** The heat plot was performed to show DEGs in diffuse type EEGC which were identified by protein mass spectrometry analysis. Treatment group was patients with positive LNM, while control group was patients with negative LNM.
