# Supplementary material for: Higher Lymph Node Metastasis Rate and Poorer Prognosis of Intestinal-Type Gastric Cancer Compared to Diffuse-Type Gastric Cancer in Early-Onset Early-Stage Gastric Cancer: A Retrospective Study
Source: Front Med (Lausanne). 2021 Dec 23;8:758977. doi: 10.3389/fmed.2021.758977 (PMC8732774; doi:10.3389/fmed.2021.758977)
Supplement: Supplementary file 6 [file Table_1.docx]

**Supplementary Table 1: Basic characteristics of patients for analysis of LNM after propensity score matching from 2010 through 2015 in SEER database**

| Variables | Total (%) | Intestinal type | Diffuse type | P Value |
| --- | --- | --- | --- | --- |
| n | 204 | 102 | 102 |  |
| **Age** |  |  |  | 0.393 |
| 20-29 | 12(5.88%) | 7(6.86%) | 5(4.9%) |  |
| 30-39 | 50(24.51%) | 21 (20.59%) | 29(28.43%) |  |
| 40-45 | 142(69.61%) | 74(72.55%) | 68(66.67%) |  |
| **Race** |  |  |  | 0.092 |
| White | 138(67.65%) | 69(67.65%) | 69(67.65%) |  |
| Black | 25(12.25%) | 17(16.67%) | 8(7.84%) |  |
| Other | 42(20.59%) | 17(16.67%) | 25(24.51%) |  |
| **Sex** |  |  |  | 1 |
| Male | 126(61.77%) | 63(61.77%) | 63(61.77%) |  |
| Female | 78(38.23%) | 39(38.23%) | 39(38.23%) |  |
| **Lymph node Metastasis** |  |  |  | 0.001 |
| No | 129(71.94%) | 53(64.98%) | 76(79.23%) |  |
| Yes | 75(28.06%) | 49(35.02%) | 26(20.77%) |  |
| **Tumor site** |  |  |  | 0.374 |
| Cardia | 43(21.08%) | 23(22.55%) | 20(19.61%) |  |
| Fundus | 11(5.39%) | 5(4.9%) | 6(5.88%) |  |
| Body | 26(12.75%) | 13(12.75%) | 13(12.75%) |  |
| Anturm | 74(36.27%) | 31(30.39%) | 43(42.16%) |  |
| Overlappping/NOS | 50(24.51%) | 30(29.41%) | 20(19.61%) |  |
| **T stage** |  |  |  | 0.762 |
| T1a | 64(31.37%) | 33(32.35%) | 31(30.39%) |  |
| T1b | 140(68.63%) | 69(67.65%) | 71(69.61%) |  |
| **Tumor Size** |  |  |  | 1 |
| ≤3cm | 0(0%) | 0(0%) | 0(0.00%) |  |
| >3cm | 204(100%) | 102(100%) | 102(100%) |  |
| **Examined LNs** |  |  |  | 0.875 |
| ≤16 | 141(69.12%) | 71(69.61%) | 70(68.63%) |  |
| >16 | 63(30.88%) | 31(30.39%) | 32(31.37%) |  |
| **Cell differentiation** |  |  |  | 0.118 |
| Well/moderately differentiated | 19(9.31%) | 11(10.78%) | 8(7.84%) |  |
| Poorly differentiated/undifferentiated | 185(90.67%) | 91(89.22%) | 94(92.16%) |  |
