# Supplementary material for: Higher Lymph Node Metastasis Rate and Poorer Prognosis of Intestinal-Type Gastric Cancer Compared to Diffuse-Type Gastric Cancer in Early-Onset Early-Stage Gastric Cancer: A Retrospective Study
Source: Front Med (Lausanne). 2021 Dec 23;8:758977. doi: 10.3389/fmed.2021.758977 (PMC8732774; doi:10.3389/fmed.2021.758977)
Supplement: Supplementary file 7 [file Table_2.docx]

**Supplementary Table 2: Basic characteristics of patients** **for analysis of survival after propensity score matching from 2010 through 2015 in SEER database**

| Variables | Total (%) | Intestinal type | Diffuse type | P Value |
| --- | --- | --- | --- | --- |
| n | 182 | 91 | 91 |  |
| **Age** |  |  |  | 0.151 |
| 20-29 | 13(7.14%） | 9(9.89%) | 4(4.40%) |  |
| 30-39 | 47(25.82%） | 19(20.88%) | 28(30.77%) |  |
| 40-45 | 122(67.03%） | 63(69.23%) | 59(64.84%) |  |
| **Race** |  |  |  | 0.8352 |
| White | 129(70.88%） | 66(72.53%) | 63(69.23%) |  |
| Black | 22(12.09%） | 11(12.09%) | 11(12.09%) |  |
| Other | 31(17.03%） | 14(15.38%) | 17(18.68%) |  |
| **Sex** |  |  |  | 1 |
| Male | 107(58.79%） | 54(59.34%) | 53(58.24%) |  |
| Female | 75(41.21%） | 37(40.66%) | 38(41.76%) |  |
| **Lymph node Metastasis** |  |  |  | *0.0576* |
| No | 116(63.74%） | 53(58.24%) | 60(65.93%) |  |
| Yes | 66(36.26%） | 49(53.85%) | 31(34.07%) |  |
| **Tumor site** |  |  |  | 0.324 |
| Cardia | 42(23.08%） | 23(25.27%) | 19(20.88%) |  |
| Fundus | 4(2.20%） | 1(1.10%) | 3(3.30%) |  |
| Body | 29(15.93%） | 12(13.19%) | 17(18.68%) |  |
| Anturm | 73(40.11%） | 28(30.77%) | 29(31.87%) |  |
| Overlappping/NOS | 34(18.68%） | 27(29.67%) | 23(25.27%) |  |
| **T stage** |  |  |  | 1 |
| T1a | 62(34.07%) | 31(34.07%) | 31(34.07%) |  |
| T1b | 120(65.93%) | 60(65.93%) | 60(65.93%) |  |
| **Tumor Size** |  |  |  | 1 |
| ≤3cm | 3(1.65%） | 2(2.20%) | 1(1.10%) |  |
| >3cm | 179(98.35%） | 89(97.80%) | 90(98.90%) |  |
| **Examined LNs** |  |  |  | 1 |
| ≤16 | 165(90.66%） | 83(91.21%) | 82(90.11%) |  |
| >16 | 17(9.34%） | 8(8.79%) | 9(9.89%) |  |
| **Cell differentiation** |  |  |  | 1 |
| Well/moderately differentiated | 13(7.14%） | 6(6.59%) | 7(7.69%) |  |
| Poorly differentiated/undifferentiated | 169(92.86%） | 85(93.41%) | 84(92.31%) |  |
