# Supplementary material for: Higher Lymph Node Metastasis Rate and Poorer Prognosis of Intestinal-Type Gastric Cancer Compared to Diffuse-Type Gastric Cancer in Early-Onset Early-Stage Gastric Cancer: A Retrospective Study
Source: Front Med (Lausanne). 2021 Dec 23;8:758977. doi: 10.3389/fmed.2021.758977 (PMC8732774; doi:10.3389/fmed.2021.758977)
Supplement: Supplementary file 8 [file Table_3.docx]

Supplementary Table 3: Intestinal type EEGC (down-regulated genes vs up-regulated genes)

| Protein ID | Gene ID |
| --- | --- |
| Q96LR5 | UBE2E2 |
| Q8TC12 | RDH11 |
| P11717 | IGF2R |
| P48960 | ADGRE5 |
| P16885 | PLCG2 |
| Q8IXJ6 | SIRT2 |
| P14209 | CD99 |
| O00399 | DCTN6 |
| P40855 | PEX19 |
| Q13428 | TCOF1 |
| Q13576 | IQGAP2 |
| O75110 | ATP9A |
| Q9Y4K0 | LOXL2 |
| P07902 | GALT |
| Q8TDL5 | BPIFB1 |
| Q14320 | FAM50A |
| Q5JRA6 | MIA3 |
| P15927 | RPA2 |
| Q9HAB8 | PPCS |
| P07948 | LYN |
| Q9UPN7 | PPP6R1 |
| Q8NBJ5 | COLGALT1 |
| P42858 | HTT |
| P09497 | CLTB |
| Q8NC56 | LEMD2 |
| Q96GF1 | RNF185 |
| Q9HD42 | CHMP1A |
| Q96HC4 | PDLIM5 |
| P19484 | TFEB |
| P11274 | BCR |
| Q9UBE0 | SAE1 |
| Q08174 | PCDH1 |
| P34913 | EPHX2 |
