# Supplementary material for: Higher Lymph Node Metastasis Rate and Poorer Prognosis of Intestinal-Type Gastric Cancer Compared to Diffuse-Type Gastric Cancer in Early-Onset Early-Stage Gastric Cancer: A Retrospective Study
Source: Front Med (Lausanne). 2021 Dec 23;8:758977. doi: 10.3389/fmed.2021.758977 (PMC8732774; doi:10.3389/fmed.2021.758977)
Supplement: Supplementary file 9 [file Table_4.docx]

Supplementary Table 4: Diffuse type EEGC (down-regulated genes vs up-regulated genes)

| Protein ID | Gene ID |
| --- | --- |
| P35237 | SERPINB6 |
| P01023 | A2M |
| P52306 | RAP1GDS1 |
| P02751 | FN1 |
| P36269 | GGT5 |
| P07686 | HEXB |
| P36955 | SERPINF1 |
| O60832 | DKC1 |
| Q9NRN5 | OLFML3 |
| Q5SYB0 | FRMPD1 |
| P27169 | PON1 |
| P51884 | LUM |
| P00751 | CFB |
| P00734 | F2 |
| P00738 | HP |
| Q9NZI6 | TFCP2L1 |
| P51888 | PRELP |
| O75400 | PRPF40A |
| P01715 | IGLV3-1 |
| P41732 | TSPAN7 |
| P01024 | C3 |
| P40426 | PBX3 |
| O75962 | TRIO |
| P49792 | RANBP2 |
| P06681 | C2 |
| P07942 | LAMB1 |
| P19827 | ITIH1 |
| Q9NYH9 | UTP6 |
| Q9UGM3 | DMBT1 |
| P01834 | IGKC |
| A0A0B4J1U7 | IGHV6-1 |
| P61626 | LYZ |
| Q6W4X9 | MUC6 |
| Q96BQ1 | FAM3D |
| Q96H79 | ZC3HAV1L |
| Q06828 | FMOD |
| Q96C23 | GALM |
| Q9BSJ8 | ESYT1 |
| Q08945 | SSRP1 |
| Q8N2S1 | LTBP4 |
| P29590 | PML |
| Q14699 | RFTN1 |
| A6NKN8 | PCP4L1 |
| Q15436 | SEC23A |
| P39060 | COL18A1 |
| Q9BWM7 | SFXN3 |
| O60568 | PLOD3 |
| Q02083 | NAAA |
| Q07954 | LRP1 |
| P04083 | ANXA1 |
| P67936 | TPM4 |
| P02788 | LTF |
| Q9Y277 | VDAC3 |
| P22352 | GPX3 |
| O95155 | UBE4B |
| P08236 | GUSB |
| Q9UBT2 | UBA2 |
| P31146 | CORO1A |
| Q9NVE7 | PANK4 |
| P19367 | HK1 |
| O43678 | NDUFA2 |
| P06576 | ATP5F1B |
| P25705 | ATP5F1A |
| P30049 | ATP5F1D |
| P17568 | NDUFB7 |
| O75146 | HIP1R |
| Q13797 | ITGA9 |
| Q9NS71 | GKN1 |
| O75363 | BCAS1 |
| P14091 | CTSE |
| P30838 | ALDH3A1 |
| P07954 | FH |
| O75521 | ECI2 |
| Q9UIJ7 | AK3 |
| P16219 | ACADS |
| Q16836 | HADH |
| P13591 | NCAM1 |
| P0DMN0 | SULT1A4 |
| P09622 | DLD |
| P10606 | COX5B |
| Q8NBF2 | NHLRC2 |
| O75390 | CS |
| P04920 | SLC4A2 |
| Q13011 | ECH1 |
| O94875 | SORBS2 |
| Q6YN16 | HSDL2 |
| P36542 | ATP5F1C |
| P28331 | NDUFS1 |
| P48047 | ATP5PO |
| P19404 | NDUFV2 |
| Q96IV0 | NGLY1 |
| Q9BSH5 | HDHD3 |
| P11310 | ACADM |
| P00813 | ADA |
| P05413 | FABP3 |
| Q86XK7 | VSIG1 |
| Q06520 | SULT2A1 |
| P13804 | ETFA |
| P36957 | DLST |
| P11177 | PDHB |
| Q92597 | NDRG1 |
| P26440 | IVD |
| O00330 | PDHX |
| P27658 | COL8A1 |
